# The effectivity of targeted therapy and immunotherapy in patients with advanced metastatic and non-metastatic cancer of the esophagus and esophago-gastric junction

**DOI:** 10.1007/s13304-022-01327-0

**Published:** 2022-07-14

**Authors:** M. J. Valkema, B. Mostert, S. M. Lagarde, B. P. L. Wijnhoven, J. J. B. van Lanschot

**Affiliations:** 1 Department of Surgery, Erasmus MC Cancer Institute, Erasmus University Medical Center Rotterdam, P.O. Box 2040, 3000 CA Rotterdam, The Netherlands; 2 Department of Medical Oncology, Erasmus MC Cancer Institute, Erasmus University Medical Center Rotterdam, P.O. Box 2040, 3000 CA Rotterdam, The Netherlands

**Keywords:** Esophageal cancer, HER2, Trastuzumab, Nivolumab, Immunotherapy, Immune checkpoint inhibitors

## Abstract

Therapies that target specific tumor drivers or immune checkpoints are increasingly explored for esophageal cancer patients. This review addresses developments in therapies with targeted anti-human epidermal growth factor receptor 2 (HER2) agents and immune checkpoint inhibitors in patients with stage IV esophageal cancer. First-line palliative treatment with the anti-HER2 agent trastuzumab in combination with chemotherapy has been approved for use in patients with HER2 positive gastro-esophageal adenocarcinoma. Neoadjuvant chemoradiotherapy plus perioperative trastuzumab however has not demonstrated a survival benefit in advanced esophageal cancer patients eligible for surgery. Potentially better responses are expected with dual agent anti-HER2 therapy instead of monotherapy. In the metastatic setting, the antibody–drug conjugate trastuzumab deruxtecan is effective after progression on trastuzumab. Nivolumab and pembrolizumab, antibodies blocking the programmed cell death 1 (PD-1) receptor on T cells, have recently gained approval for clinical use in esophageal cancer patients for specific indications. Synergistic effects might be achieved with combinations of immune checkpoint inhibitors that target PD-1 on T cells or PD ligand 1 (PD-L1) on tumor cells and anti-cytotoxic T-lymphocyte-associated antigen 4 (CTLA-4) receptor on T cells. Multiple clinical trials investigating combinations of targeted and immunotherapies, with or without (neo)adjuvant chemo(radio)therapy, for curative and palliative treatment, are underway, and are expected to deliver a long-awaited improvement in the prognosis of esophageal cancer patients.

## Introduction

Despite advances in therapies over the past years, esophageal cancer remains an aggressive disease. Especially in patients with stage IV disease the survival is poor with a 5-year overall survival of less than 5% [1, 2].

Stage IV esophageal cancer is subdivided in stages IVa and IVb [3]. Stage IVa entails more than six positive locoregional lymph nodes without distant metastases (T1-4/N3/M0), irrespective of tumor histology. Stage IVa also includes T1-4a/N2/M0 in adenocarcinomas, and T4/N0-2/M0 in squamous cell carcinomas. When distant metastases (M1) are present, the disease is classified as stage IVb.

There are several treatment options for advanced esophageal cancer. The first-choice for locally advanced cancer is neoadjuvant chemotherapy (nCT) or chemoradiotherapy (nCRT) followed by surgery. Neoadjuvant treatment according to the ChemoRadiotherapy for Oesophageal cancer followed by Surgery Study (CROSS) has demonstrated a survival benefit which persists after 10-year follow-up [4]. In patients in whom nCRT followed by surgery is expected to be non-curative, as can be the case in unresectable bulky primary tumors or lymph node metastases outside the planned radiotherapy or surgical field, treatment with induction chemotherapy can be considered. Induction chemotherapy with paclitaxel and carboplatin or paclitaxel and cisplatin is able to downstage the tumor, achieving a resectable tumor in some patients [5].

New combinations of (neo)adjuvant therapies are explored to improve treatment efficacy in patients with stage IV esophageal cancer. Ongoing research focuses for example on the tolerability of a sequential combination of chemotherapy with fluorouracil, leucovorin, oxaliplatin and docetaxel (FLOT) [6] and chemoradiotherapy (nCRT according to CROSS) [7], to downstage oligometastatic esophageal cancer and optimize locoregional disease control [8, 9].

For stage IVb disease various systemic regimens are used for palliation. Most commonly, a platinum compound (oxaliplatin, carboplatin or cisplatin) is combined with 5-fluorouracil (5-FU) or capecitabine or a taxane [10]. Regardless of the choice of regimen, the beneficial effect on overall survival is small, especially for squamous cell carcinoma. For patients with adenocarcinoma of the gastro-esophageal junction (GEJ), more effective treatment regimens are available and they can be treated with three subsequent lines of therapy.

Moving beyond conventional chemotherapy, targeted therapy and immunotherapy are employed to control and diminish tumor growth in a more subtle manner. Targeted therapy inhibits tumor driver mutations or overexpressed antigens, such as human epidermal growth factor receptor 2 (HER2), vascular endothelial growth factor (VEGF) or transforming growth factor-β (TGF-β) receptor. Immunotherapy unleashes the immune system’s innate antitumor immune response by targeting specific pathways of the immune system. Targeted therapy and immunotherapy are widely applied in other cancer types, such as anti-HER2 therapy in breast cancer [11] and immunotherapy with the monoclonal antibody durvalumab against programmed cell death ligand 1 (PD-L1) in non-small cell lung cancer [12]. This review addresses developments regarding HER2-targeted therapies and immunotherapies and discusses the potential role of these therapies to improve treatment response and survival in patients with stage IV esophageal cancer.

### Anti-HER2 therapy

**Fig. 1:**
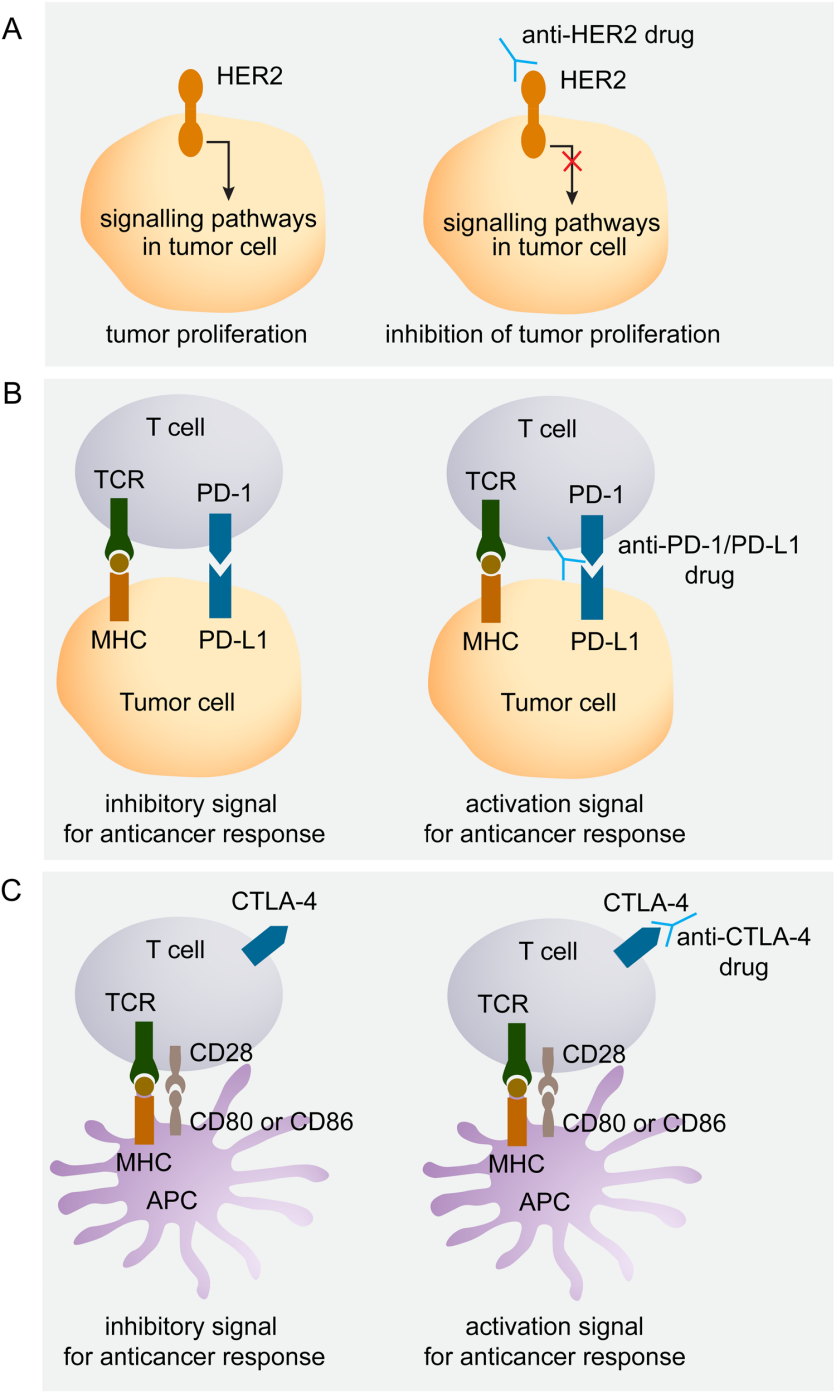
Schematic overview of targets involved in tumor proliferation or immune regulation. **A)** a tumor cell is shown expressing HER2. HER2 activation leads to activation of intracellular stimulating pathways for tumor proliferation. By blocking HER2 with an anti-HER2 agent such as trastuzumab, downstream signaling and tumor proliferation is prevented. **B)** a tumor cell connects with a T cell through the T cell receptor (TCR) and major histocompatibility complex (MHC). Connection of PD-L1 with PD-1 downregulates the immune response. Blocking the PD-L1/PD-1 connection with for example nivolumab may lead to stimulation of an anticancer response. **C)** an antigen presenting cell (APC) such as a dendritic cell (shown in the figure) connects with a T cell through MHC-TCR interaction and co-stimulating signals between CD28 on the T cell and CD80 or CD86 on the APC. This upregulates expression of CTLA-4 on T cells (left side of Fig. 1c) which can downregulate a T cell response when interacting with CD80 and CD86 molecules (not shown in figure). Blocking CTLA-4 by for instance ipilimumab allows activation of a T cell response. *APC* antigen presenting cell, *HER2* human epidermal growth factor receptor 2, *PD-1* programmed cell death protein 1, *PD-L1* programmed cell death protein ligand 1, *TCR* T cell receptor, *MHC* major histocompatibility complex, Source figure: own source

Amplification of *ERBB2,* the gene encoding for HER2 (also known as HER2/neu), or HER2 overexpression is present in approximately 30% of esophageal adenocarcinomas [13, 14]. HER2 is one of the family of tyrosine protein kinases, including HER1 (the protein encoded by *EGFR*), HER2, HER3 and HER4. Upon activation of the HER2 protein, multiple intracellular signaling pathways can be initiated, which promote tumor proliferation (Fig. 1a). Trastuzumab is an example of a human recombinant monoclonal antibody that binds to the extracellular part of the HER2 protein to prevent intracellular signaling [11].

#### Metastatic cancer

In 2010, the ToGA trial was the first to show a survival benefit with trastuzumab in the palliative setting of patients with HER2 positive adenocarcinoma of the stomach or GEJ [15]. Patients who received anti-HER2 therapy in combination with standard chemotherapy had slightly better survival than patients who received chemotherapy alone (median overall survival 13.8 months versus 11.1 months, hazard ratio (HR) 0.74, 95% CI 0.60–0.91, P = 0.0046). Treatment with trastuzumab was well tolerated. Palliative first-line treatment with chemotherapy and trastuzumab has been adopted in the guidelines for metastatic gastro-esophageal adenocarcinoma with overexpression of HER2 [16]. Still, variation between hospitals exists in whether HER2 status is assessed in these patients [14].

Pertuzumab, an anti-HER2 monoclonal antibody that binds to another domain of HER2 than trastuzumab, has been demonstrated to improve survival when it was added to treatment with trastuzumab and chemotherapy in breast cancer patients [17, 18]. Accordingly, it was hypothesized that pertuzumab plus trastuzumab and chemotherapy might improve outcomes in esophago-gastric cancer. However, the phase III JACOB trial could not demonstrate a statistically significant survival benefit in patients who received pertuzumab plus trastuzumab and chemotherapy versus patients who received placebo plus trastuzumab and chemotherapy in the first-line treatment of metastatic HER2 positive GEJ or gastric adenocarcinoma [19].

Within the palliative setting, therapies with antibody–drug conjugates (ADCs) such as trastuzumab deruxtecan are of interest. Trastuzumab deruxtecan is a drug that has trastuzumab bound to a cytotoxic topoisomerase I inhibitor through a tetrapeptide-based linker, and thus combines anti-HER2 therapy with cytotoxic therapy, which is only delivered in the proximity of HER2 expression, into one agent. In the third-line treatment of patients with HER2-positive metastatic gastric or GEJ adenocarcinoma encouraging results were published [20]. The phase III DESTINY-Gastric04 trial (NCT04704934) now investigates trastuzumab deruxtecan in patients with HER2-positive unresectable or metastatic gastric or GEJ adenocarcinoma who have disease progression after first-line therapy with trastuzumab (Table 1). Accrual of patients is ongoing.

**Table 1:** Overview of ongoing phase III clinical trials on immunotherapy in esophageal cancer as identified on clinicaltrials.gov. (Search August 30th 2021: “esophageal cancer”, “phase III”, “recruiting”, “not yet recruiting”, “active, not recruiting”. Only recruiting phase II/III studies have been included.)

	Registration number clinicaltrails.gov (name trial)	Phase	Treatment	Target (agent)	Patient population	Location of participating sites
Single agent immunotherapy	NCT03748134 (ORIENT-15)	III	CT + sintilimab CT + placebo	PD-1 (sintilimab)	unresectable or metastatic ESCC	Globally
	NCT03777657	III	CT + tislelizumab CT + placebo	PD-1 (tislelizumab)	unresectable or metastatic gastric or GEJ adenocarcinoma	Globally
	NCT03783442	III	CT + tislelizumab CT + placebo	PD-1 (tislelizumab)	unresectable or metastatic ESCC	Globally
	NCT03829969	III	CT + toripalimab CT + placebo	PD-1 (toripalimab)	unresectable or metastatic ESCC	China
	NCT03957590	III	dCRT + tislelizumab dCRT + placebo	PD-1 (tislelizumab)	unresectable ESCC	China
	NCT04210115 (KEYNOTE-975)	III	dCRT + pembrolizumab dCRT + placebo	PD-1 (pembrolizumab)	unresectable EAC or ESCC	Globally
	NCT04280822	III	nCT + adjuvant toripalimab nCT	PD-1 (toripalimab)	resectable ESCC	China
	NCT04404491	III	nCRT + adjuvant camrelizumab nCRT + placebo	PD-1 (camrelizumab)	ESCC with local recurrence after surgery	China
	NCT04426955	III	dCRT + camrelizumab dCRT + placebo	PD-1 (camrelizumab)	unresectable ESCC	China
	NCT04540211 (SKYSCRAPER-08)	III	CT + tiragolumab + atelizumab CT + placebo	TIGIT (tiragolumab) PD-L1 (atelizumab)	unresectable or metastatic ESCC	Multiple countries in Asia
	NCT04543617 (SKYSCRAPER-07)	III	tiragolumab + atelizumab tiragolumab placebo + atelizumab tiragolumab placebo + atelizumab placebo	TIGIT (tiragolumab) PD-L1 (atelizumab	unresectable ESCC without progression after dCRT	Globally
	NCT04550260 (KUNLUN)	III	dCRT + durvalumab dCRT + placebo	PD-L1 (durvalumab)	unresectable ESCC	Globally
	NCT04807673 (KEYSTONE-002)	III	nCT + neoadjuvant pembrolizumab + adjuvant pembrolizumab nCT	PD-1 (pembrolizumab)	resectable ESCC	China
	NCT04821778	III	dCRT + anti-PD-1/PD-L1 or nimotuzumab dCRT	HER1 (nimotuzumab) PD-1 or PD-L1 (not reported)	unresectable ESCC	China
	NCT04821843	III	nCT + adjuvant anti-PD-1/PD-L1 or nimotuzumab and CT nCRT + adjuvant anti-PD-1/PD-L1 or nimotuzumab and CT	HER1 (nimotuzumab) PD-1 or PD-L1 (not reported)	resectable ESCC	China
	NCT04848753	III	nCT + neoadjuvant toripalimab nCT + placebo	PD-1 (toripalimab)	resectable ESCC	China
Combination therapy	NCT02872116 (Checkmate 649)	III	nivolumab + ipilimumab*	PD-1 (nivolumab) CTLA-4 (ipilimumab)	unresectable or metastastic non-HER2-positive adenocarcinoma of the esophagus, GEJ or stomach	Globally
	NCT03604991	II/III	nCRT + adjuvant nivolumab and ipilimumab nCRT + adjuvant nivolumab nCRT + neoadjuvant nivolumab + adjuvant nivolumab and ipilimumab nCRT + neoadjuvant nivolumab + adjuvant nivolumab	PD-1 (nivolumab) CTLA-4 (ipilimumab)	resectable adenocarcinoma esophagus or GEJ	USA
	NCT03615326 (KEYNOTE 811)	III	CT + pembrolizumab + trastuzumab CT + placebo + trastuzumab	PD-1 (pembrolizumab) HER2 (trastuzumab)	unresectable or metastatic HER2 positive gastric or GEJ adenocarcinoma	Globally
	NCT04499924 (MOUNTAINEER-02)	II/III	CT + tucatinib + trastuzumab + ramucirumab CT + tucatinib placebo + trastuzumab placebo + ramucirumab CT + tucatinib + trastuzumab placebo + ramucirumab	HER2 (tucatinib) HER2 (trastuzumab) VEGFR-2 (ramucirumab)	unresectable or metastatic HER2 positive gastric or GEJ adenocarcinoma	Globally
	NCT04704934 (DESTINY-Gastric04)	III	trastuzumab deruxtecan ramucirumab + paclitaxel	antibody–drug conjugate (trastuzumab deruxtecan) tyrosine kinase inhibitor (remucirumab)	unresectable or metastatic HER2 positive gastric or GEJ adenocarcinoma with progression after anti-HER2 therapy	Globally
	NCT04879368 (INTEGRATEIIb)	III	regorafenib + nivolumab CT	tyrosine kinase inhibitor (regorafenib) PD-1 (nivolumab)	metastatic AC or undifferentiated carcinoma of the GEJ or stomach	Globally
	NCT04949256	III	pembrolizumab + lenvatinib + CT pembrolizumab + CT	PD-1 (pembrolizumab) tyrosine kinase inhibitor (lenvatinib)	metastatic ESCC	Globally

*CT* chemotherapy, *dCRT* definitive chemoradiotherapy, *EAC* esophageal adenocarcinoma, *EGFR* epidermal growth factor receptor, *ESCC* esophageal squamous cell carcinoma, *GEJ* gastro-esophageal junction, *HER2* human epidermal growth factor receptor 2, *ITIM* immunoreceptor tyrosine-based inhibitory motif, *nCRT* neoadjuvant chemoradiotherapy, *nCT* neoadjuvant chemotherapy, *PD-1* programmed cell death protein 1, *PD-L1* programmed cell death protein ligand 1, *RT* radiotherapy, *TIGIT* T cell immunoreceptor with immunoglobin and ITIM domain, *VEGF* vascular endothelial growth factor receptor

^*^Single treatment arm of which the full results [41] are not yet available; results of other study arms have been published[26]

#### Advanced non-metastatic cancer

In the neoadjuvant setting, anti-HER2 therapy is also increasingly investigated. The phase III RTOG 1010 trial demonstrated no statistically significant benefit in overall survival, progression-free survival or pathologically complete response rate with trastuzumab added to chemoradiotherapy [21]. The trial included 203 patients with HER2-positive adenocarcinoma of the esophagus or GEJ. Patients were randomized between chemoradiotherapy (carboplatin/paclitaxel with concurrent 50.4 Gy radiotherapy) followed by surgery and chemoradiotherapy combined with peri-operative trastuzumab. The authors suggested that the negative results might be attributed to the degree of HER2 expression in patients selected for the study. The measured HER2 expression at baseline might not have been representative of the total tumor HER2 expression due to tumor heterogeneity or loss of HER2 expression over time. Furthermore, it was suggested that trastuzumab might be more effective in combination with another chemotherapy regimen.

Despite negative results of the JACOB trial, a synergistic effect is expected when dual agent anti-HER2 therapy is administered, potentially leading to better clinical outcomes than with single agent therapy in a curative setting. The ongoing randomized phase II INNOVATION study (NCT02205047) [22] (results not yet available) and the already published TRAP study [23] investigate this topic. In the TRAP study, 40 patients with HER2-positive adenocarcinoma received nCRT according to CROSS in combination with trastuzumab and pertuzumab administered over a period of 13 weeks. Neoadjuvant treatment was well tolerated, with 83% of patients completing neoadjuvant therapy. Ninety-five percent of patients underwent surgery; all had radical resections. In 34% of patients a pathologically complete response was observed. At a median follow-up of 32 months, the 3-year overall survival rate was 71%. In comparison with a propensity score-matched historical cohort of patients having received CROSS, HR for risk of death was 0.58 (95% CI 0.34–0.97) with HER2-targeted therapy. The randomized phase III TRAP2-trial is expected to start recruiting in the Netherlands shortly, a trial that will hopefully lead to more robust evidence for the effectiveness of anti-HER2 therapies in the treatment of esophageal cancer.

### Mechanisms of immunotherapy

In a normal physiological situation, there is a balance between inhibition and stimulation of the immune system. At immune checkpoints, inhibitory signals are put in place to prevent auto-immune disease. One of the mechanisms tumors can exhibit to escape from an immunologic anti-tumor response, is to express ligands that interact with these immune checkpoints [24, 25]. Hence, one of the many approaches in the field of immunotherapy is to target immune checkpoints. Important immune checkpoint inhibitors are antibodies blocking programmed cell death ligand 1 (PD-L1) on the surface of tumor cells (expressed by 15–40% of esophageal cancers [26–28]), antibodies targeting programmed death-1 (PD-1) receptor on the surface of T cells and antibodies targeting cytotoxic T-lymphocyte-associated antigen 4 (CTLA-4) receptor on the surface of T cells. In this review, the scope regarding immunotherapy is limited to these three targets, as most data have been generated with these compounds in esophageal and gastric cancer.

Tumors that express PD-L1 can interact with PD-1 receptors on T cells, thereby inhibiting the anti-tumor immune response (Fig. 1b). By blocking either PD-L1 or PD-1, T cells are activated and the tumor is subjected to a T cell mediated immune response. Durvalumab and atezolizumab are examples of anti-PD-L1 drugs [29]. Nivolumab and pembrolizumab are examples of anti-PD-1 monoclonal antibodies. PD-L1 expression is thought to be a predictor of response to therapy, but its ability to predict anti-tumor response is still topic of research. The CTLA-4 receptor, which can be upregulated by T cells, is another target for immune checkpoint inhibitors. CTLA-4 is involved in T cell suppression where it interacts with antigen presenting cells (Fig. 1c). Blockage of CTLA-4 through for example ipilimumab consequently promotes T cell activation.

Immunotherapy is associated with a broad range of side-effects, as it can cause auto-immune like inflammation in any organ or tissue in the body. The most common side-effects of immunotherapy are nonbacterial colitis, skin rash, hypophysitis, pneumonitis and hepatitis [26, 27, 30], which often need treatment with immune suppressors such as corticosteroids.

### Single agent immunotherapy

The effectivity of immune therapy is thought to be at least partly dependent on the number of tumor antigens that are presented to the immune system. When more genetic variations such as mutations are present, there are more neoantigens for the immune system to be recognized as foreign. The number of mutations per megabase genome is referred to as the tumor mutational burden (TMB). Esophageal cancer is one of the cancer types with the highest TMBs, not far behind melanoma, for example [31].

Nonetheless, in contrast to melanoma, results of clinical trials with immunotherapy in esophageal cancer patients have been disappointing thus far. The effectivity of immune therapy is likely driven by a complex system involving multiple factors, both tumor- and patient-specific, which we are just beginning to understand. Based on recently published trials, however, immunotherapy is a promising treatment option in esophageal cancer patients.

#### Metastatic cancer

The Checkmate 649 trial has investigated the use of nivolumab in the first-line palliative setting for patients with HER2-negative, unresectable or metastatic adenocarcinoma of the esophagus and/or stomach. In this trial, 1581 patients were randomized between chemotherapy plus nivolumab versus chemotherapy alone [26]. Approximately 70% of patients had gastric cancer. Chemotherapy consisted of oxaliplatin and capecitabine or fluorouracil. All patients, regardless of PD-L1 expression, were eligible for randomization. Co-primary endpoints were overall and progression-free survival in patients with a high PD-L1 Combined Positive Score (CPS), which is a measure of the number of PD-L1 positive cells relative to all viable tumor cells. In patients with PD-L1 CPS ≥ 5, which involved 60% of all patients, a survival benefit of 3.3 months was seen in patients receiving nivolumab (HR 0.71, 98.4% CI 0.59–0.86, P < 0.0001). Patients with PD-L1 CPS < 5 did not show convincing benefit (P for interaction 0.011). More grade 3 or 4 adverse were seen in the nivolumab plus chemotherapy arm as compared to the chemotherapy alone group (59% versus 44%, respectively), but the overall safety and tolerability profile was acceptable. Based on the results of the Checkmate 649 trial, nivolumab plus chemotherapy as a first-line palliative treatment for HER2-negative adenocarcinoma with PD-L1 CPS ≥ 5 has been approved for clinical use by the Food and Drug Administration (FDA) and the European Medicines Agency (EMA).

In the setting of unresectable or metastastic esophageal squamous cell cancer, refractory or resistant to first-line palliative treatment, nivolumab as a single agent has been evaluated in the ATTRACTION 3 trial [32]. The ATTRACTION-3 phase III trial randomized 419 patients at 90 participating hospitals in the USA, Europe and Asia. An improvement in median overall survival of 2.5 months was observed in patients who received nivolumab, in comparison with patients who received chemotherapy with paclitaxel or docetaxel (HR 0.77, 95% CI 0.62–0.96, P = 0.019). Progression-free survival was not significantly different between the treatment arms, and PD-L1-expression was not predictive of response. Patients in the nivolumab arm experienced less toxicity than patients in the chemotherapy group (18% versus 63% grade 3 or 4 adverse events, respectively). While the overall survival benefit was statistically significant and the FDA and EMA have approved the use of nivolumab for this indication, the clinical significance of this limited effect on overall survival without effect on progression-free survival should be weighed against its safety profile and costs.

Apart from nivolumab, many other checkpoint inhibitors are topic of research for patients in a palliative setting (Table 1). Pembrolizumab, which targets PD-1, has been investigated as second-line palliative therapy in esophageal cancer patients in the KEYNOTE-181 phase III trial [33]. A statistically significant improvement in median overall survival of 2.6 months was seen in patients with CPS score ≥ 10 who received pembrolizumab, as compared to patients who received investigator’s choice chemotherapy. More recently, the results of the KEYNOTE-590 phase III trial have been published [34]. In this trial, patients with previously untreated, locally unresectable or metastatic adenocarcinoma or squamous cell carcinoma of the esophagus or GEJ were randomized between pembrolizumab plus chemotherapy and placebo plus chemotherapy. Therapy with pembrolizumab was shown to improve progression-free survival in the complete group of 749 randomized patients. Patients with squamous cell carcinoma and CPS ≥ 10 showed the greatest improvement in median overall survival (2.8 months and 4.1 months increase in median overall survival, respectively). Following these results, the FDA and EMA have approved pembrolizumab in combination with chemotherapy in the first-line setting for advanced esophageal cancer with CPS ≥ 10.

#### Advanced non-metastatic cancer

Following the results of the Checkmate 577 trial, nivolumab has gained approval by the FDA and EMA for clinical use in esophageal cancer patients after neoadjuvant chemoradiation and radical resection. Adjuvant nivolumab was administered to patients without a pathologically complete response in their resection specimen for the duration of one year [27]. After a follow-up of 24.4 months, median disease-free survival was 22.4 months in the nivolumab group and 11.0 months in the placebo group (HR for disease recurrence or death 0.69, 96.4% CI 0.56 – 0.86, P < 0.001). PD-L1 expression in pre-treatment biopsies, categorized as < 1% or ≥ 1%, did not predict outcome. Nivolumab was well tolerated, as in nine percent of patients in the nivolumab group experienced grade 3–4 adverse events leading to discontinuation of treatment, as opposed to three percent in the placebo group. Reports of long-term survival outcomes are not yet available, but based on these first results it is likely that nivolumab will become part of standard of care in many countries.

Patients with a pathologically complete response to neoadjuvant chemoradiation were not included in the CheckMate 577 trial, and will not have access to immune therapy in the non-metastatic setting. To better select patients who will benefit from esophageal resection, efforts are ongoing to optimize and test “surgery as needed” strategies. In the Surgery As Needed for Oesophageal cancer (SANO) -1 trial, patients with a clinically complete response as assessed at 12 weeks after neoadjuvant chemoradiation were randomized to undergo resection or active surveillance [35]. While the results of this study are awaited, active surveillance as an alternative treatment strategy is offered to an increasing number of patients. To optimize treatment for these patients with a clinically complete response after neoadjuvant chemoradiation, in the multicenter SANO-3 trial patients will be treated with maintenance nivolumab for the duration of one year.

A summary of ongoing phase III trials that investigate the addition of immune therapy to neoadjuvant chemoradiation and peri-operative chemotherapy is shown in Table 1. Moreover, many interesting phase II trials are underway for patients in a curative setting. The ongoing phase II TAPESTRY trial (NCT04595149) investigates the potential of bintrafusp alfa in combination with definitive chemoradiotherapy in squamous cell carcinoma. Bintrafusp alfa is an agent that binds to transforming growth factor-β (TGF-β) receptor, involved in cell growth, as well as to PD-L1. Thus, it exerts a simultaneous inhibitory function to both TGF-β and PD-L1. For locally advanced squamous cell esophageal cancer, the PALACE-2 trial (NCT04435197) is ongoing. This trial investigates pembrolizumab in combination with nCRT according to CROSS. Earlier results of a small phase Ib trial have shown that this regimen was well-tolerated and achieved a pathologically complete response in 56% (10/18) of patients. The trial is currently proceeding as a phase II study.

### Combination therapies

Immune checkpoint inhibition might be more effective when multiple checkpoints are targeted. Dual blockade of PD-1 or PD-L1 ánd CTLA-4 is thought to achieve a synergistic effect, potentially leading to better outcomes [29]. Dual immunotherapy might be advantageous in the treatment of tumors with low PD-L1 expression. In the first-line treatment of pleural mesothelioma, the administration of ipilimumab (blocking CTLA-4) in combination with nivolumab (blocking PD-1) has recently shown a statistically significant improvement in 3-year overall survival (Checkmate 743 trial) [36]. In contrast, earlier studies in this disease with single agent immunotherapy reported disappointing results [37, 38]. Of note, the toxicity of combined immunotherapy vastly exceeds that of single-agent therapy, resulting in almost half of patients experiencing grade 3 or 4 adverse events (compared to 17% in the single-agent arm) [39].

For patients with previously untreated locally unresectable or metastatic esophageal squamous cell carcinoma, the results of the randomized phase III Checkmate 648 trial have been published [40]. The three study arms of this trial were as follows: 1) nivolumab with chemotherapy (fluorouracil and cisplatin), 2) nivolumab plus ipilimumab without chemotherapy, and 3) chemotherapy alone. Patients in any of the two immunotherapy arms had significantly superior overall survival compared to the chemotherapy arm (median overall survival per arm: 1) 13.2 months, 2) 12.7 months and 3) 10.7 months). Especially patients with PD-L1 expression ≥ 1% seemed to benefit from immunotherapy (median overall survival per arm: 1) 15.4 months, 2) 13.7 months and 3) 9.1 months). The trial was not powered to evaluate effectiveness between nivolumab plus chemotherapy versus nivolumab and ipilimumab without chemotherapy. More grade 3 or 4 adverse events were seen in the nivolumab plus chemotherapy arm (47%) compared to the nivolumab plus ipilimumab arm (32%) and the chemotherapy alone arm (36%). Nivolumab plus chemotherapy and nivolumab plus ipilimumab in the first-line treatment of esophageal squamous cell cancer with ≥ 1% PL-L1 expression has recently gained approval for clinical use.

Several trials investigating the use of dual immunotherapy in esophageal cancer are underway (Table 1). The aforementioned Checkmate 649 trial also incorporated a study arm in which HER2-negative adenocarcinoma patients received nivolumab plus ipilimumab. First results reported at a median follow-up of 35 months indicate that there is no statistically significant improvement in overall survival as compared to the chemotherapy alone arm [41]. The full publication on the updated results is still awaited.

The French phase II CRUCIAL trial (NCT03437200) investigates nivolumab plus ipilimumab in combination with definitive chemoradiotherapy. Another phase Ib trial has shown that the combination of durvalumab (anti-PD-L1) and tremelimumab (anti-CTLA-4) plus platinum-based chemotherapy is tolerable in patients with esophageal squamous cell carcinoma [42]. The trial has been extended to further investigate the efficacy and safety of this combination therapy.

In addition to combining two immunotherapies, several phase II studies are underway that combine anti-HER2 therapy with immune checkpoint inhibitors [43]. For example, the phase III KEYNOTE 811 study (NCT03615326) is conducted globally and includes patients with previously untreated, locally unresectable or metastatic gastric or GEJ adenocarcinoma. Patients in this trial are randomized for chemotherapy with trastuzumab (anti-HER2) and pembrolizumab (anti-PD-1), or chemotherapy with trastuzumab and placebo. Results of the first 264 included patients after a median follow-up of 12 months show a statistically significant increase in response according to RECIST criteria and an increase in duration of response in patients in the experimental arm [44]. Results at a longer follow-up need to be awaited.

## Outlook

A challenge remains the selection of patients who will benefit from targeted therapies and immunotherapies. Better patient selection would prevent exposure of patients to ineffective, toxic and costly regimens. With regard to anti-HER2-targeted therapy, expression levels of HER2 as well as growth factor-binding protein 7 (Grb7) have been suggested as interesting potential prognostic biomarkers for further research [23].

Observations with respect to the tumor micro-environment (TME) might also help to refine patient selection or to find new targets for treatment. In the PERFECT feasibility trial for example, atezolizumab, an anti-PD-L1 drug, was investigated in 40 patients in combination with nCRT according to CROSS [45]. Administration of atezolizumab was feasible, but no statistically significant differences were observed in overall survival or progression-free survival in comparison with a historical CROSS cohort. In subgroup analyses, tumor resistance mechanisms were explored. The immune system of non-responders was characterized by exhausted T cells and low numbers of cytotoxic T cells. In responders, however, high interferon-ɣ (IFNɣ) expression, a protein which leads to upregulation of PD-L1, was shown. Biomarkers for response should be incorporated in clinical trial designs for instance as stratification factors, to increase our ability to select patients most likely to benefit from a specific therapy. Moreover, new drugs or combinations of drugs could be developed for those patients not benefitting from current therapies.

## Conclusion

Targeted therapies with anti-HER2 agents and/or checkpoint inhibitors have started to show encouraging results in the treatment of esophageal cancer patients within phase II and III clinical trials. In patients with potentially curable disease as well in more advanced settings, many clinical trials are ongoing to investigate combinations of chemotherapy, radiotherapy, (dual) anti-HER2 therapies and (dual) checkpoint inhibitors. As for now, nivolumab is the first immune checkpoint inhibitor being approved by the EMA for clinical use in an adjuvant setting for patients with residual esophageal cancer after neoadjuvant therapy. Indications for nivolumab in the palliative setting have been rapidly expanding. These indications include nivolumab plus chemotherapy in the first-line treatment of HER2-negative adenocarcinoma with CPS ≥ 5 and squamous cell carcinoma with ≥ 1% PD-L1 expression, nivolumab plus ipilimumab in the first-line treatment of squamous cell carcinoma with ≥ 1% PD-L1 expression, and nivolumab monotherapy in the second-line treatment of squamous cell carcinoma. Pembrolizumab plus chemotherapy has been approved for clinical use in the first-line treatment of metastatic esophageal cancer with CPS ≥ 10. Upcoming trials will hopefully be able to further expand tailored treatment options. Taking into consideration that these treatments are toxic and have a marked impact on society in terms of their costs, finding better predictive biomarkers is pivotal.
